# Application of a deep learning image reconstruction (DLIR) algorithm in head CT imaging for children to improve image quality and lesion detection

**DOI:** 10.1186/s12880-021-00637-w

**Published:** 2021-07-08

**Authors:** Jihang Sun, Haoyan Li, Bei Wang, Jianying Li, Michelle Li, Zuofu Zhou, Yun Peng

**Affiliations:** 1 Imaging center, Beijing Children’s Hospital, Capital Medical University, National Center for Children’s Health, No. 56, Nanlishi Road, Xicheng District, Beijing, 100045 China; 2 GE Healthcare, Milwaukee, WI USA; 3 Stanford University, Stanford, CA USA; 4 Department of Radiology, Fujian Provincial Maternity and Children’s Hospital, Affiliated Hospital of Fujian Medical University, No. 18 Daoshan Road, Gulou District, Fujian, 350000 China

**Keywords:** CT, Head, Children, Low-dose, IR, Deep learning

## Abstract

**Background:**

To evaluate the performance of a Deep Learning Image Reconstruction (DLIR) algorithm in pediatric head CT for improving image quality and lesion detection with 0.625 mm thin-slice images.

**Methods:**

Low-dose axial head CT scans of 50 children with 120 kV, 0.8 s rotation and age-dependent 150–220 mA tube current were selected. Images were reconstructed at 5 mm and 0.625 mm slice thickness using Filtered back projection (FBP), Adaptive statistical iterative reconstruction-v at 50% strength (50%ASIR-V) (as reference standard), 100%ASIR-V and DLIR-high (DL-H). The CT attenuation and standard deviation values of the gray and white matters in the basal ganglia were measured. The clarity of sulci/cisterns, boundary between white and gray matters, and overall image quality was subjectively evaluated. The number of lesions in each reconstruction group was counted.

**Results:**

The 5 mm FBP, 50%ASIR-V, 100%ASIR-V and DL-H images had a subjective score of 2.25 ± 0.44, 3.05 ± 0.23, 2.87 ± 0.39 and 3.64 ± 0.49 in a 5-point scale, respectively with DL-H having the lowest image noise of white matter at 2.00 ± 0.34 HU; For the 0.625 mm images, only DL-H images met the diagnostic requirement. The 0.625 mm DL-H images had similar image noise (3.11 ± 0.58 HU) of the white matter and overall image quality score (3.04 ± 0.33) as the 5 mm 50% ASIR-V images (3.16 ± 0.60 HU and 3.05 ± 0.23). Sixty-five lesions were recognized in 5 mm 50%ASIR-V images and 69 were detected in 0.625 mm DL-H images.

**Conclusion:**

DL-H improves the head CT image quality for children compared with ASIR-V images. The 0.625 mm DL-H images improve lesion detection and produce similar image noise as the 5 mm 50%ASIR-V images, indicating a potential 85% dose reduction if current image quality and slice thickness are desired.

## Background

**Fig. 1:**
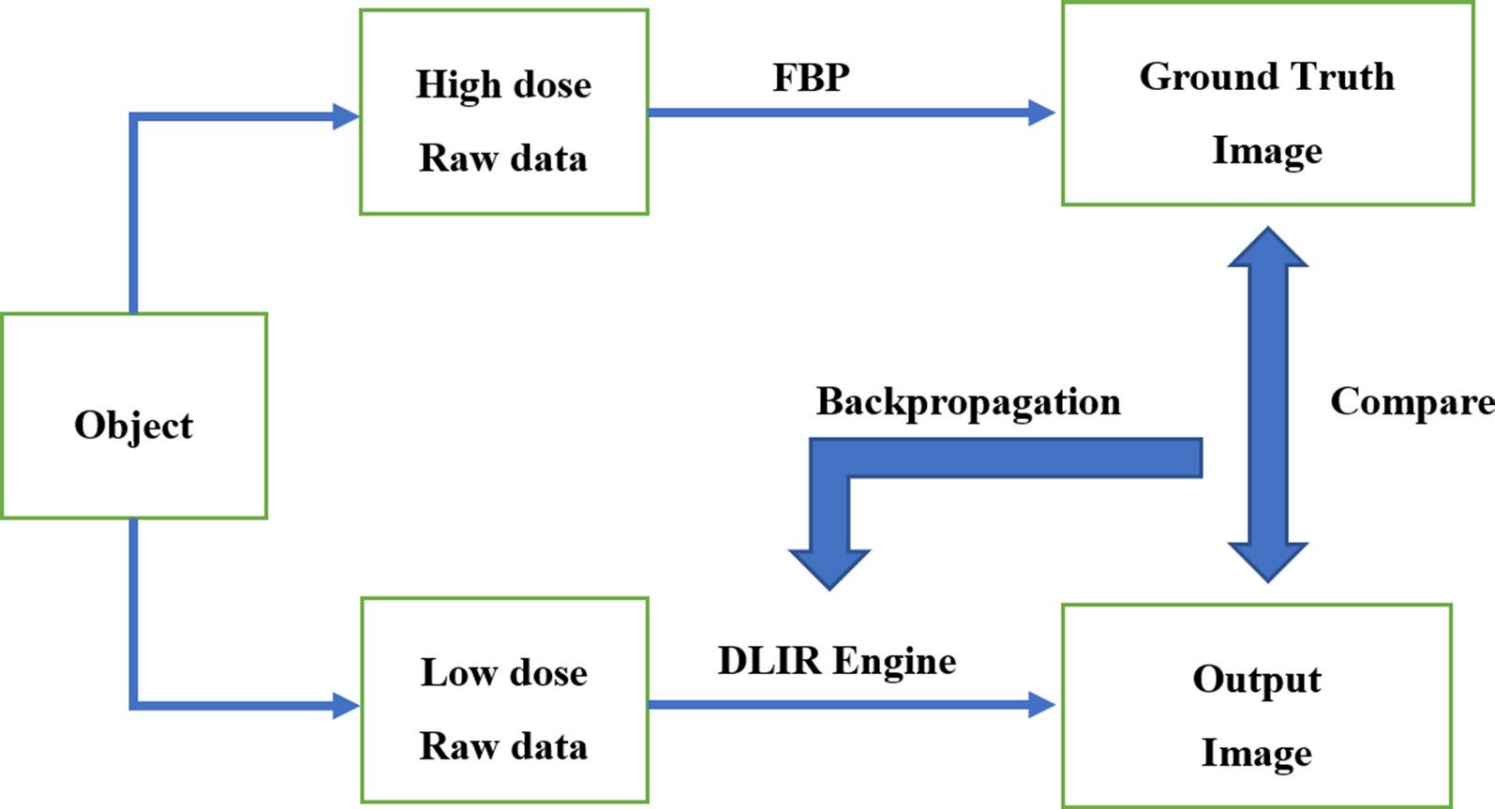
A schematic of training the DLIR algorithm

CT examination is fast and non-invasive and is often used to image the head for injury and convulsion in pediatric emergency care [1–5]. However, in pediatric CT imaging, how to minimize the CT dose and make it fit in better with the as low as reasonably achievable (ALARA) principle is always a topic worth discussing [6]. There are several reports calling for proper indication selection [7, 8], in order to reduce the total number of head CT, and modified scanning protocol for head CT [9–13] to minimize the radioactive damage in children. One of the effective and proven methods in CT imaging is to use iterative reconstruction algorithms to reduce image noise in low radiation dose CT scans to maintain image quality [13, 14], and the ability of Iterative reconstruction (IR) to reduce image noise is often converted to dose reduction in clinical applications to maintain similar image noise. However, iterative reconstruction algorithms have also shown their limitations, especially in balancing noise (dose) reduction and image texture and overall image quality. Recently, a deep learning image reconstruction (DLIR) algorithm (Commercial name, TrueFidelityTM, GE Healthcare, Waukesha USA), a deep neural network-based recon engine, has been proposed to address challenges of iterative reconstruction algorithms. DLIR features a deep neural network (DNN), which was trained with high quality FBP data sets to learn how to differentiate noise from signals, and to intelligently suppress the noise without impacting anatomical and pathological structures [15]. The DLIR engine builds upon specific knowledge of the detailed design of the particular CT system which includes knowledge of the conditioning of the collected data. Even more importantly, this knowledge is embedded within a DNN, which is capable of learning through a large number of real-world examples. Through these examples, the DLIR engine gradually optimizes the coefficients of its internal network as it figures out how to arrive at the optimal solution (i.e. the best image) by comparing against the ground truth training data sets. Once the DLIR engine has been trained and fully tested, the inference network uses the trained coefficients to deploy the new image reconstruction in a clinical environment (Fig. 1). Therefore, the objective of our paper was to retrospectively review a series of head CT images reconstructed using the newly developed DLIR algorithm from children who received emergency care and to evaluate whether the application of this DLIR algorithm could further improve the image quality and therefore make it possible to further reduce the dose of head CT for children or significantly improve spatial resolution and lesion detectability while maintaining low radiation dose, in comparison with the state-of-the-art adaptive statistical iterative reconstruction (ASIR-V, GE Healthcare, Waukesha USA) algorithm.

## Methods

### Patients information

This was a retrospective study approved by the Ethical Committee of our Hospital for evaluating image quality, and patient informed consent was waived. All Head CT scans were performed in accordance with the approved guidelines and regulations of our hospital. The CT scans were acquired consecutively from 2019–10–08 to 2019–10–31 for children who had a head trauma, convulsion or other mental symptoms or requested by emergency doctors to exclude intracranial abnormalities.

### Scan parameters

Scans were performed on a 256-row detector CT scanner (Revolution CT, GE Healthcare) using an axial scan mode with the 12 cm, 14 cm or 16 cm detector coverage based on patient head size. The tube voltage of 120 kVp was used with 0.8 s gantry rotation time. The tube current was adjusted base on children’s age in the range from 150 to 210 mA: 150 mA for 0–2 years old, 170 mA for 3–6 years old, 190 mA for 7–12 years old and 210 mA for 13 years and older. The radiologic technologists could adjust the tube current by ± 10 mA based on their own experience. All scans were acquired in a single rotation with patients in a fixed position. For those children who were too young to cooperate, sedation with oral chloral hydrate (10%, 0.4 ml/Kg) was applied before the scanning.

The raw data were reconstructed into images of two different slice thicknesses, 5 mm and 0.625 mm, using the standard reconstruction kernel and 4 different reconstruction algorithms: the traditional filtered back projection (FBP), 50% adaptive statistical iterative reconstruction-V (50%ASIR-V), 100%ASIR-V and DLIR with high setting (DL-H). The 50%ASIR-V images used the standard 50% weight in the ASIR-V algorithm and were served as the reference standard in clinical practice. The 100%ASIR-V used a 100% weight to further reduce image noise. The volumetric CT dose index (CTDIvol) and dose length product (DLP) for each patient scan were recorded.

### Image quality assessment

All images were transmitted to a GE AW4.7 CT workstation (GE Healthcare). All children's private information and reconstruction parameters were hidden during the evaluation process, and the observers can adjust the window width and window level to the position deemed appropriate. Two pediatric radiologists (with 15 years and 10 years of diagnostic experience) evaluated the image quality according to the scoring standard individually. In cases where different scores were given a unified result was achieved after discussion. If no agreement could be achieved, a senior neuroradiologist (with 25 years of diagnostic experience) was asked to give a final result.

The image qualities evaluated included the subjective scoring and objective measurement.

The subjective image quality was evaluated using a 1–5 scoring system, with 3 stands for a satisfying imaging quality. Subjective evaluation included the clarity of sulci/cisterns, the boundaries between the white and gray matters, and the overall image quality. Score 1, nondiagnostic, severe image noise, no visualization of sulci/cisterns, cannot define boundaries between white and gray matters; 2, only enough for detection, still not fully diagnostic, heavy image noise, no clear visualization of sulci/cisterns, no clear boundaries between white and gray matters; 3, diagnostic with good image quality, moderate image noise and somewhat blurred boundaries, fully measurable); 4, diagnostic with very good image quality, little image noise, clear identification for sulci/cisterns, clear boundaries between white and gray matters; and 5, diagnostic with excellent image quality, absence of image noise, very clear identification for sulci/cisterns, sharp boundaries between white and gray matters. The overall image quality was scored mainly based on the physician’s confidence to make diagnosis using the image. Meanwhile, the number of detected lesions was counted in each reconstruction group, including hemorrhagic lesion, encephaledema, cerebral hernia, encephalomalacia, intracranial pneumatosis, broadening of ventricles and cisterns, immature parenchymal hypodensity and soft tissue lesion in the scalp. The number of lesions was counted based on every intact boundaries, symmetric broadening of ventricles, general broadening of cisterns and immature parenchymal hypodensity in each patient.

After the subjective evaluation, the two radiologists performed the objective quantification on the AW workstation together. For each patient, a ROI was placed on the basal ganglia slice to measure the density of gray and white matters. Specifically, the ROI was placed on the uniform parts in the center of the head of the left caudate nucleus and in the frontal lobe on the same layer to measure the CT values and the associated standard deviation (SD) of gray and white matters, respectively. The signal-to-noise ratio (SNR) of gray and white matters, the contrast-noise-ratio (CNR) between gray and white matters were calculated using the following formula: SNR = CT value/SD, CNR = 2*[(CT value (gray matter) − CT value (white matter))]/(Image noise(gray matter) + Image noise(white matter)).

All the data were represented as mean ± SD. Continuous variables following the normal distribution were analyzed by using the repeated measures analysis of variance with Bonferroni correction. The ordinal scales or variables that failed to follow normal distribution were analyzed by using Friedman's test. Images with the same slice thickness were initially analyzed. The differences between the 5 mm 50%ASIR-V images and 0.625 mm DL-H images were also analyzed to investigate the dose saving potential using DLIR with the image noise level and 5 mm slice thickness currently accepted clinically. *P* < 0.05 was considered statistically significant.

## Results

**Fig. 2:**
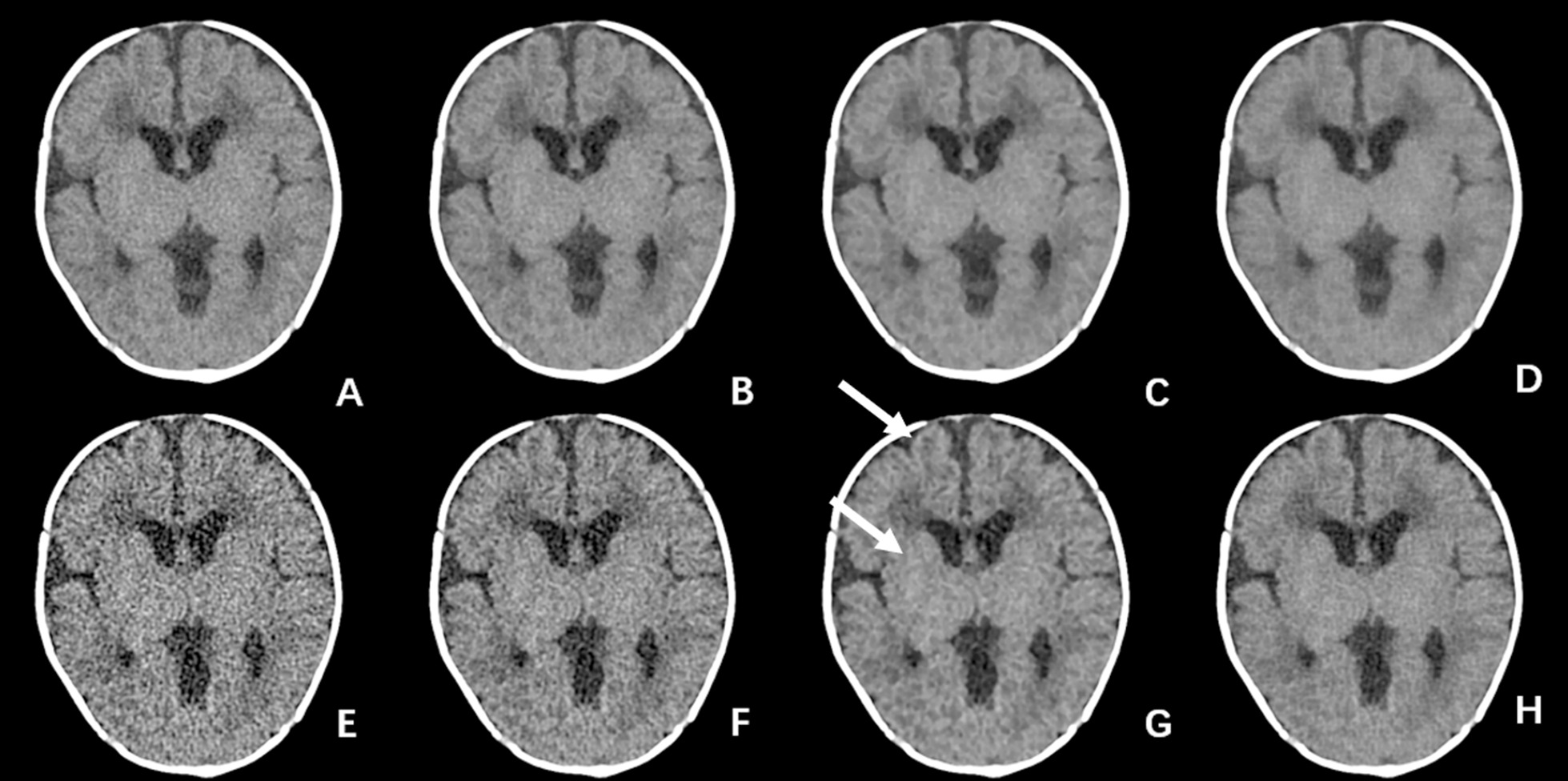
An infant with fever for 18 days and diagnosis of purulent meningitis. The scanning tube current was 150 mA, CTDI = 15.26 mGy, DLP = 183.21 mGy cm, **A**–**D** were 5 mm images with FBP, 50%ASIR-V, 100%ASIR-V and DL-H, respectively, **E**–**H** were 0.625 mm images. It can be observed that with the increase of ASIR-V weight, the noise of both the thick images and thin images decreased, but the blurred margins in 100% ASIR-V images were more pronounced (white arrows), and the subjective scores for the edges of gray and white matters were not improved. While the noise in DL-H images was reduced, the sharpness of edge maintained compared to FBP images, which is more suitable for observation

Fifty children (35 males and 15 females) were included. The median age was 2.0 years old (0.1–14 years), The tube current, CTDIvol, and DLP for the present study was 173.91 ± 17.34 mA, 18.18 ± 2.82 mGy and 269.43 ± 57.95 mGy cm, respectively. The subjective evaluation results are shown in Table 1. As expected, under the low dose scan condition, even at the standard 5 mm slice thickness, FBP reconstructions failed to fully meet the diagnostic requirement with high image noise. On the other hand, at 5 mm slice thickness, the 50%ASIR-V, 100%ASIR-V and DL-H all met the diagnostic requirement, except the overall image quality of 100%ASIR-V, where blotchy or plastic looking images were observed. DL-H had the highest scores in all aspects of the subjective evaluation. There were statistically significant differences in the clarity for sulci/cisterns, boundary between white and gray matters, and overall image quality between 50%ASIR-V and DL-H, and in the overall image quality between 100%ASIR-V and DL-H (all *p* < 0.05). For the 0.625 mm images, only DL-H reconstructions met the diagnostic requirement. There was no significant difference in the image noise measurement and subjective image quality score between the 0.625 mm DL-H images and 5 mm 50%ASIR-V images (all *p* > 0.05) (Fig. 2). All SPSS results are listed in a supplementary file.

**Table 1:** Subjective evaluation of different reconstruction algorithms

	FBP	50%ASIR-V	100%ASIR-V	DL-H	*F* value	*P* value
5 mm images						
SC	2.47 ± 0.50	3.04 ± 0.19	3.53 ± 0.50*	3.71 ± 0.46*	116.00	< 0.001
BM	2.25 ± 0.44	3.04 ± 0.19	3.51 ± 0.50*	3.67 ± 0.51*	131.68	< 0.001
WQ	2.25 ± 0.44	3.05 ± 0.23*	2.87 ± 0.39*	3.64 ± 0.49	129.57	< 0.001
0.625 mm images						
SC	1.51 ± 0.54	2.13 ± 0.55	2.93 ± 0.47*	3.15 ± 0.45*	143.20	< 0.001
BM	1.05 ± 0.23	1.78 ± 0.42	2.44 ± 0.50	3.02 ± 0.36	146.06	< 0.001
WQ	1.04 ± 0.19	1.82 ± 0.39	2.42 ± 0.50	3.04 ± 0.33	148.45	< 0.001

SC: Clarity level of sulci/cisterns boundaries; BM: boundaries between the white and gray matters; WQ: whole image quality

*Without significant statistical differences for the numbers with * between the paired comparison

**Fig. 3:**
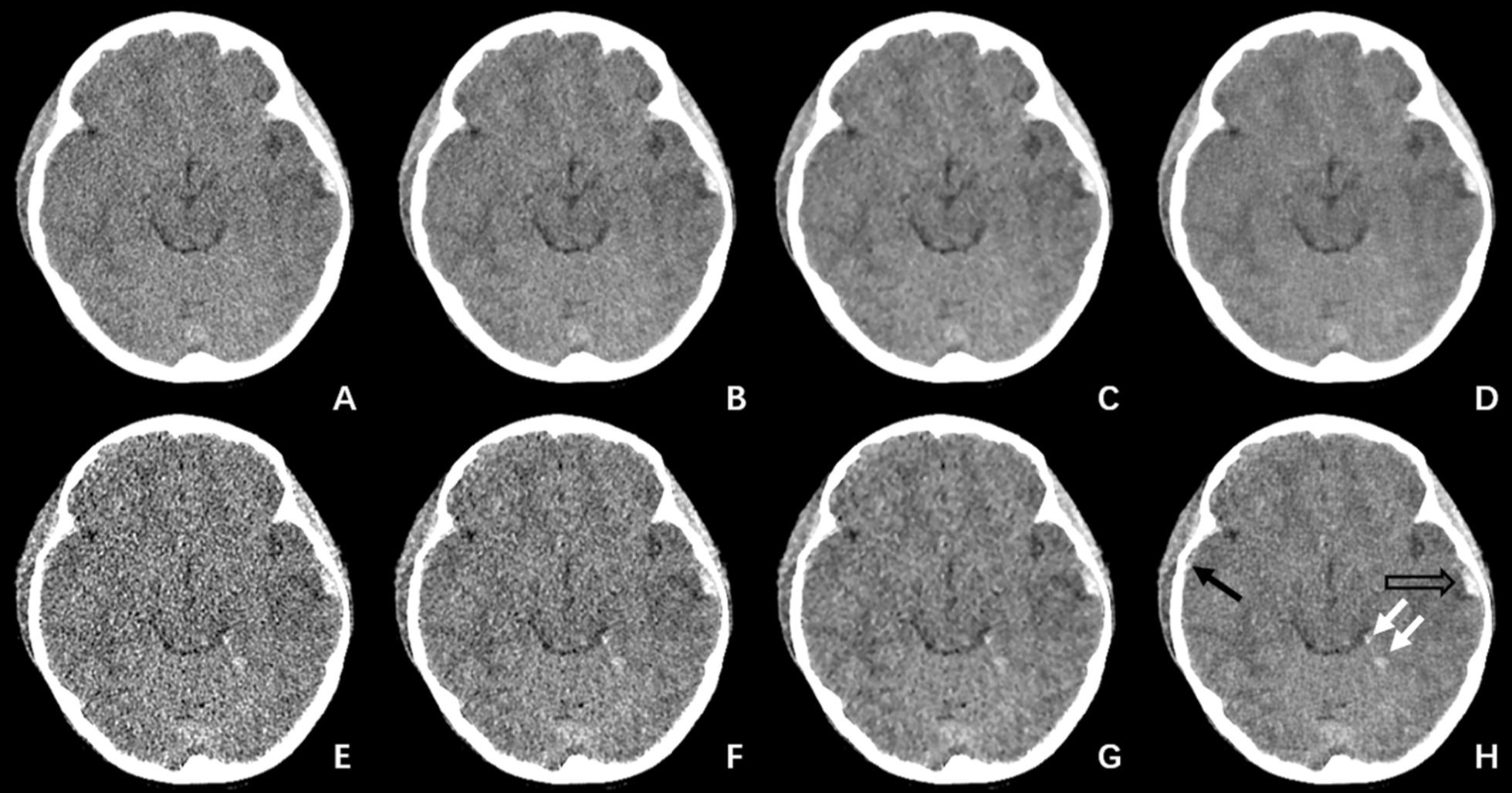
A child under the age of 8, 6 h after suffering from traffic accident. The scan voltage was 120 kV, current was 175 mA, CTDI = 17.43 mGy, DLP = 278.88 mGy cm, **A**–**D** were 5 mm images and **E**–**H** were 0.625 mm images with FBP, 50%ASIR-V, 100%ASIR-V and DL-H, respectively. Hemorrhage and peripheral edema of left temporal were detected (black hollow arrow) and could be seen on all images, 3C and 3D had the lowest image noise. Only thin slice images could show the epidural hemorrhage (black arrow) and hemorrhages in tentorium cerebellum (white arrows), 100%ASIR-V and DL-H could confirm it, but 100%ASIR-V had blurred margins, couldn’t depict the lesion exactly. Only DL-H thin slice image met the diagnostic requirement

Sixty-five lesions were identified in 26 patients using the 5 mm images of all reconstruction algorithms, including 18 cases of hemorrhagic lesion, 5 encephaledema, 4 immature parenchymal, 5 cerebral hernia, 16 dilation of ventricles/cisterns, 5 encephalomalacia, 4 intracranial pneumatosis and 8 soft tissue swelling. The thin-layer images improved the detection for hemorrhagic lesions by 2–4 cases than the 5 mm images. The 100%ASIR-V and DL-H detected the highest number of lesions at 69 (Fig. 3), however, DL-H had much higher confidence level as indicated by the overall image quality score of the 0.625 mm images (Tables 1 and 2).

**Table 2:** Number of detected lesions with different reconstruction algorithms

Algorithm	Total	Hemo-rrhage	Encepha-ledema	Immature paren-chymal	Cerebral hernia	Dilation of ventricles/cisterns	Encepha-lomalacia	Intracranial pneu-matosis	Soft tissue swelling
5 mm FBP	65	18	5	4	5	16	5	4	8
5 mm 50%	65	18	5	4	5	16	5	4	8
5 mm 100%	65	18	5	4	5	16	5	4	8
5 mm DL-H	65	18	5	4	5	16	5	4	8
0.6 mm FBP	67	20	5	4	5	16	5	4	8
0.6 mm 50%	67	20	5	4	5	16	5	4	8
0.6 mm 100%	69	22	5	4	5	16	5	4	8
0.6 mm DL-H	69	22	5	4	5	16	5	4	8

The quantitative measurement was shown in Table 3. The CT values were not significantly different across all images. It was shown that DL-H and 100%ASIR-V had the lowest level of noise and the highest level of SNR and CNR among images of the same thickness. There was no significant difference in SD, SNR and CNR values among the DL-H and 100%ASIR-V for the 5 mm and 0.625 mm images.

**Table 3:** Objective measurement of different reconstruction algorithms

	FBP	50% ASIR-V	100% ASIR-V	DL-H	*F*	*P*
5 mm images						
GM CT	32.58 ± 2.19	32.74 ± 2.36	32.77 ± 2.33	32.46 ± 2.31	0.23	0.88
GM SD	4.51 ± 1.15	3.35 ± 0.84	2.28 ± 0.62*	2.26 ± 0.50*	93.83	< 0.001
WM CT	25.75 ± 2.66	25.49 ± 2.82	25.52 ± 2.84	25.56 ± 2.73	0.10	0.96
WM SD	4.28 ± 0.84	3.16 ± 0.60	2.21 ± 0.57	2.00 ± 0.34	159.18	< 0.001
GM SNR	7.67 ± 1.83	10.35 ± 2.44	15.40 ± 4.29*	15.03 ± 3.13*	82.28	< 0.001
WM SNR	6.20 ± 1.12	8.28 ± 1.46	12.05 ± 2.42	13.07 ± 2.24	159.09	< 0.001
CNR	1.61 ± 0.60	2.30 ± 0.79	3.33 ± 1.10*	3.30 ± 1.06*	46.08	< 0.001
0.625 mm images						
GM CT	32.57 ± 2.62	32.71 ± 2.68	32.76 ± 2.57	32.64 ± 2.52	0.06	0.98
GM SD	8.23 ± 1.68	5.74 ± 1.18	3.43 ± 0.80*	3.35 ± 0.68*	221.42	< 0.001
WM CT	25.36 ± 3.00	25.37 ± 3.06	25.42 ± 3.00	25.47 ± 2.85	0.02	1.00
WM SD	8.07 ± 1.57	5.60 ± 1.13	3.38 ± 0.81*	3.11 ± 0.58*	247.66	< 0.001
GM SNR	4.11 ± 0.86	5.94 ± 1.31	10.09 ± 2.53*	10.09 ± 1.96*	157.54	< 0.001
WM SNR	3.24 ± 0.67	4.68 ± 0.97	7.86 ± 1.80	8.40 ± 1.45	201.80	< 0.001
CNR	0.91 ± 0.41	1.33 ± 0.55	2.21 ± 0.85*	2.26 ± 0.77*	54.59	< 0.001

SG: clarity level of sulci/cisterns boundaries; BM: boundaries between the white and gray matters; WQ: whole image quality

*Without significant statistical differences for the numbers with * between the paired comparison

## Discussion

In our study, we evaluated a deep learning image reconstruction (DLIR) algorithm in its ability to significantly improve image quality at the same slice thickness and maintain similar image noise at much thinner slice thickness for potential dose reduction as the state-of-the-art 50%ASIR-V algorithm and demonstrated the improved lesion detection with thinner slice in head CT.

CT is commonly used in pediatric emergency care, and the head CT is often the first choice for skull fracture, deformation and trauma complicated with hemorrhage [1, 6, 16]. Although some guidelines have pointed out that CT is not necessary for children with the first episode of convulsion [17], physicians still use it in emergency care to examine lesions in the brain due to its efficiency and convenience. With the more frequent use of CT comes the increased request for dose reduction to minimize radiation damage [18, 19].

One of the effective methods for reducing radiation dose in CT is the use of iterative reconstruction algorithms. Iterative reconstruction (IR) algorithms can improve the quality of images scanned in low signal cases [20] and therefore make it possible to reduce the radiation dose. Some of the proven IR algorithms include the adaptive statistical iterative reconstruction (ASIR-V, GE Healthcare), advanced modeled iterative reconstruction (ADMIRE, Siemens) and Iterative Model Reconstruction (IMR, Philips). The effectiveness of these algorithms in improving the image quality in the head CT for children have been reported [14, 21–23]. It has been reported that compared with the traditional FBP reconstruction, ASIR-V can reduce image noise for brain by 60% [14], ADMIRE can improve SNR for the white matter by 58.59% [22] and IMR can improve SNR for the white matter by 92.86% [23]. In our study, we reconstructed both thin- and thick-slice images using FBP, 50%ASIR-V, 100%ASIR-V and DLIR in the high setting (DL-H) to assess the differences of different reconstruction algorithms. Confirming the previous study, our results demonstrated that the 100%ASIR-V algorithm could improve SNR for the white matter by 94.35% in the 5 mm images and 142.59% in the 0.625 mm images. Our results also suggested that DL-H could further improve the image quality by significantly reducing the noise level in both thin- and thick-slice images (Fig. 3). Compared with the traditional FBP reconstruction, DL-H improved SNR for the white matters by 110.81% in the 5 mm images and 159.26% in the 0.625 mm images compared with the traditional FBP. DL-H significantly reduced image noise in the gray matter by 50% and 33% in the 5 mm images and 59% and 42% in the 0.625 mm images compared with FBP and 50%ASIR-V, respectively.

We further compared the performance between DL-H and ASIR-V with the maximum weighting (100%ASIR-V). Although the objective quantification showed a comparable noise reduction between 100%ASIR-V and DL-H in both 5 mm and 0.625 mm thickness images, DL-H was superior to 100%ASIR-V in the subjective evaluation. The 100%ASIR-V demonstrated blotchy appearance in the images with some blurred edges compared to DL-H and failed to fully satisfy diagnostic requirements with the 5 mm and 0.625 mm 100%ASIR-V images. We believe this has something to do with how the current IR algorithms are designed. IR algorithms adopt the gradual solution method, through the preset reconstruction models, and iterate to find the optimal solution that can match the input data. To minimize the modeling complexity, simplified models are often used with limited parameters (generally less than 100). Because the limitation of modelling and simplification of the model complexity, although the image noise is reduced, the plastic-looking image artifacts are produced, resulting in image edge blurring. This is common for regular IR algorithms. Despite of the differences in the principles of IR algorithms from various manufacturers, the plastic-looking artifact exists and is enhanced with the increased weight of IR [24, 25], which impacts the subjective evaluation from the observers. In a latest phantom study, Greffier et al. [26] found that the ASIR-V algorithm would lower the noise power spectrum (NPS) peaks during image reconstruction, especially when 100% ASIR-V is used. However, high spatial frequency signals are also reduced in this process resulting in lower NPS average spatial frequency when 100%ASIR-V is used. The reduction of the overall image noise at the cost of losing certain spatial resolution and detection rate are often seen in other regular IR algorithms [13, 22, 23].

The image noise reduction ability of IR algorithms is often hard to be directly related to dose reduction potential due to limitations of only scanning patient once to minimize patient dose. In this study, we simulated lower dose scans by comparing images of different slice thickness. Since the X-ray flux used to reconstruct images is directly proportional to the image slice thickness, the signals used for the 0.625 mm images are only one eighth of those for the 5 mm images, an about 85% signal reduction or x-ray dose reduction. However, our results indicated that even at one eighth of the signal strength, the image noise for the 0.625 mm DL-H images was statistically the same as the 5 mm 50%ASIR-V images and the subjective image quality scores were similar between the two types of images (3.05 ± 0.23 for the 5 mm 50%ASIR-V images vs. 3.04 ± 0.33 for the 0.625 mm DL-H images). The fact that the image noise level and image quality of the 5 mm 50%ASIR-V images were acceptable for current routine clinical use suggested that one might be able to save a significant radiation dose (up to 85%) by using DL-H algorithm to maintain the current noise level and 5 mm slice thickness. Of course, if thinner slices were desirable, then dose reduction potential would be reduced.

In clinical application, thinner image slice is more desirable since the thin-slice images enhance the spatial resolution and provide more information. However, in the conventional CT imaging, it is a balance between radiation dose and spatial resolution. Thinner slice typically means higher noise level and lower image quality. In order to maintain reasonable image noise under low radiation dose, 5 mm images are often used for diagnosis at a cost of losing some of the disease relevant information. This information loss could also impact the detection of lesions, especially the detection for small or subtle lesions. In our study, we found that both the 100%ASIR-V and DL-H could detect 4 more lesions in 4 patients with the thinner slice images (Fig. 3), all of which were small hemorrhagic lesions (with sizes less than 3.0 mm), which amounted to 8% reduction in missed diagnosis. On the other hand, related to the lower NPS spatial frequencies, lesions on 100%ASIR-V images were blurred with less confidence, and the precise size of lesions could not be measured, which were reasons why the 100%ASIR-V was not fully accepted for detecting and characterizing small lesions in our study. For larger lesions, such as ventricle dilation, soft tissue lesions in the scalp, immature parenchymal hypodensity, brain hernia, or for lesions with uneven density distribution such as intracranial pneumatosis and large hemorrhagic lesions, even though the thin-layer images did not improve the detection rate, they provided more comprehensive information and clearer boundaries for more confidence diagnosis and more accurate estimation of the lesion range and volume. The application of DL-H algorithm in the 0.625 mm images clearly demonstrated the brain structures as well as the number and the range of the lesions. Such comprehensive information provided precise evidence for further diagnosis and treatment.

Our study could be improved from the following aspects. First, the sample size was limited since we could only use the raw data stored in the revolution scanner for DLIR reconstruction. Therefore, only the emergency cases were included. Secondly, we only compared the quality of images processed by different algorithms and the lack of comparison between our results with the pathological gold standard prevented us from accurately evaluating the difference between the estimated and the actual hemorrhagic amount. Nonetheless, the head CT has been widely accepted in clinical practice, and the CT feature comparison in our study was of high confidence. Thirdly, this was a retrospective study where the same raw data were reconstructed into images with different thickness by various algorithms to simulate signal reduction. Although we showed that DLIR could obtain similar image noise and image quality score as the current state-of-the-are reconstruction at one eighth of the slice thickness, we did not prospectively reduce radiation dose at the time of patient scanning. Further investigation is needed to compare the image quality when the radiation dose is reduced. Finally, we did not evaluate the skull due to its high density and the high resolution for fracture lines. Previous studies have shown the evaluation of skull under low radiation dose. Thus, we did not include the skull in this study.

## Conclusion

DLIR at the high setting (DL-H) significantly reduces image noise and improves the quality of the images without the negative blurring artifacts as seen in the regular IR-processed images with high weights. The 0.625 mm thin slice DL-H images improve lesion detection and produce similar image noise compared with the routine 5 mm 50%ASIR-V images. This could be used in two ways in pediatric CT: significantly improving spatial resolution and lesion detection ability at the similar low radiation dose levels as the current ones; or providing potential dose reductions up to 85% if the current image noise level and spatial resolution are acceptable.

## Data Availability

The datasets used and/or analyzed during the current study available from the corresponding author on reasonable request.

## Abbreviations

ALARA: As low as reasonably achievable
ASIR-V: Adaptive statistical iterative reconstruction-v
CNR: Contrast-to-noise ratio
CTDIvol: CT dose index
DLIR: Deep learning image reconstruction
DL-H: DLIR with a high setting
DLP: Dose length product
FBP: Filtered back-projection
IR: Iterative reconstruction
NPS: Noise power spectrum
SD: Standard deviation
SNR: Signal-to-noise ratio
